# Short‐term resource allocation during extensive athletic competition

**DOI:** 10.1002/ajhb.23052

**Published:** 2017-10-10

**Authors:** Daniel P. Longman, Sean P. Prall, Eric C. Shattuck, Ian D. Stephen, Jay T. Stock, Jonathan C. K. Wells, Michael P. Muehlenbein

**Affiliations:** ^1^ Department of Archaeology and Anthropology University of Cambridge Cambridge United Kingdom; ^2^ Laboratory for Evolutionary Medicine, Department of Anthropology University of Texas at San Antonio, San Antonio, Texas; ^3^ Department of Psychology Macquarie University Sydney Australia; ^4^ UCL Institute of Child Health Childhood Nutrition Research Centre London United Kingdom; ^5^ Department of Anthropology Western University, London Ontario N6A 3K7 Canada; ^6^ Laboratory for Evolutionary Medicine Department of Anthropology, Baylor University

## Abstract

**Objectives:**

Following predictions from life history theory, we sought to identify acute trade‐offs between reproductive effort (as measured by psychological arousal) and somatic maintenance (via functional measures of innate immunity) during conditions of severe energetic imbalance.

**Methods:**

Sixty‐six male ultramarathon runners (ages 20 to 37 years) were sampled before and after a lengthy race. Saliva and sera were collected for testosterone and immunological analyses (hemolytic complement activity and bacterial killing ability). Lean body mass was assessed by bioelectrical impedance, and libido was measured using a slideshow of arousing and neutral images.

**Results:**

Following predictions, there was a significant decrease in salivary testosterone levels (109.59 pg/mL versus 97.61 pg/mL, *P* < .001) and arousal scores in response to provocative images (5.40 versus 4.89, *P* = .001) between prerace and postrace time points. Additionally, participant bacterial killing ability (*P* = .035) and hemolytic complement activity (*P* = .021) increased between prerace and postrace.

**Conclusions:**

Decreased libido and testosterone with concomitant heightened innate immune responses suggest a shift in energetic priorities away from reproduction and toward maintenance/defense during a period of energetic stress.

## 1 INTRODUCTION

Life history theory predicts the existence of trade‐offs between competing physiological functions relating to reproduction, maintenance, defense, growth, and storage under conditions of limited environmental resources (Reznick, Nunney, & Tessier, 2000; Stearns, 1992). Individuals that have developed effective mechanisms for both acquisition and optimal allocation of energy in their particular ecological niche are thereby advantaged (Angiletta, Wilson, Navas, & James, 2003; Lotka, 1922). According to evolutionary theory, every individual organism should exhibit behavior intended to enhance genetic contributions to subsequent generations. However, a life history strategy involving a greater allocation of resources toward reproduction necessitates a reduction in the resources available for other functions. Consequently, an individual may allocate energy to traits which enhance fecundity and fertility or invest in traits enhancing survivorship.

Fluctuations in the availability of environmental energy influence the relative investment in reproduction versus survivorship. This is exemplified by nutritional infecundability, whereby a negative shift in the relative balance between energy intake and expenditure adversely affects the fecundity in a range of mammalian species (Bronson, 1991; Mosley, 2012; Wade, Schneider, & Li, 1996). Despite the pathological connotations, it is likely that conditions of sub‐optimal nutrition will be encountered at some point during the lifetime of a mammal, and animals will have been selected to protect functions necessary for survival at the expense of less essential processes. Thus, available energy is partitioned according to a set of priorities that maximize the chance of survival and thereby optimize long term reproductive success (Wade, 1992).

The principle of allocation proposes the existence of negative correlations between investment in reproduction and somatic traits such as maintenance, growth, or storage (Cody, 1966; Roff, 1992: Zera & Harshman, 2001). Despite the intuitive appeal of a stereotypical life history trade‐off featuring a negative relationship between two traits, such negative covariation in traits are frequently absent when phenotypic comparisons are made between individuals within a population (Glazier, 2000). Furthermore, negative correlations are less likely to be reported in a field setting than in a tightly controlled laboratory study. This may be explained by inter‐individual variation in resource acquisition exceeding variation in resource allocation (Van Noordwijk & de Jong, 1986).

Identifying quasi‐experimental scenarios in which negative correlations are observable in the field would break new ground in the study of human life history trade‐offs. While one cannot control individual energy intake in the field without compromising ecological validity, it may be possible to nullify the effect of this variation in resource acquisition by experimentally controlling energy balance. It is not the absolute values of energy intake and expenditure which force the individual to make resource allocation decisions, but the imbalance of the two (Bronson, 1991). Therefore, if the energy deficit is high enough, variation in food intake between individuals will be inconsequential; all individuals will be energetically stressed.

This study uses male participants from running ultramarathons to investigate the relative investment between reproduction and survivorship. Ultramarathons, defined as races lasting over 4 hours (Knez, Coombes, & Jenkins, 2006) (but often several days in length). Recent work suggests that high levels of energy expenditure cause metabolic adaptation to reduce total energy output (Pontzer et al., 2016), and the high energetic costs of locomotion necessitated by ultramarathons are expected to necessitate physiological trade‐offs. This is due to energy deficit induced by a combination of the high levels of energy expenditure in physiological systems linked to locomotion, and the lack of opportunity to ingest any significant meals (Knechtle & Bircher, 2005; Knechtle, Enggist, & Jehle, 2005).

Testosterone functions as a physiological regulator, influencing the allocation of energetic resources between life history traits (Hau, 2007; Muehlenbein & Bribiescas, 2005). From a physiological perspective, testosterone plays a significant role in stimulating muscle growth (Kadi, 2008), which is beneficial in both inter‐sexual (Dixson, Halliwell, East, Wignarajah, & Anderson, 2003; Fan, Dai, Liu, & Wu, 2005; Frederick & Haselton, 2007; Lavrakas, 1975) and intra‐sexual (Bribiescas, 2001; Dijkstra & Buunk, 2002) selection. Testosterone is also implicated in behavioral aspects of human male reproductive effort. Sex drive, or libido, is a vitally important motivational force in human behavior (Ariely & Loewenstein, 2006; Bancroft, 1988; Darwin, 1871). In addition to its role in generating sexual motivation (Baumeister, Catanese, & Vohs, 2001; Beach, 1976; Sherwin, 1988), testosterone is important in modulating qualities believed to be beneficial in the male mating effort, such as confidence and assertiveness in social situations (Bagatell, Matsumoto, Christensen, Rivier, & Bremner, 1993; Elias, 1981; Ellison, 2003; Morley, 2003). Consequently, we measured testosterone levels, lean body mass, and libido of participants.

Measures of investment in immune function, a proxy for the body's investment in survivorship, were taken through the use of two functional assays of innate immune responses (bacteria killing assay and hemolytic complement assay). The purpose of the bacteria killing assay is to measure the functional ability of integrative immunological components, including opsonizing proteins and antibodies, to lyse a known quantity of *Echerichia coli* bacteria relative to a positive control (Muehlenbein, Prall, & Chester, 2011). The hemolytic complement assay serves to measure the ability of the antibody‐dependent pathway of the complement system to lyse pathogens. These assays assess innate immune responses only. While a comprehensive assessment of immunocompetence would require examination of the adaptive immune response as well, these functional assays are arguably more relevant than simple measures of inflammation (e.g., C‐reactive protein or sIgA), and meaningful results have previously been reported in humans and nonhuman primates (Georgiev, Muehlenbein, Prall, Emery Thompson, & Maestripieri, 2015; Prall & Muehlenbein, 2014; Prall & Muehlenbein, 2015; Prall et al., 2015).

This article aims to build upon current knowledge of human male energetic investment in reproduction and survivorship through analysis of male ultramarathon runners. While past studies predominantly consider trade‐offs acting on a long‐term evolutionary scale, the present study employs the conceptual framework provided by life history theory as a reference to consider acute trade‐offs. It was hypothesized that markers of investment in reproductive function (Demas, Zysling, Beechler, Muehlenbein, & French, 2011) will decrease in magnitude, allowing for an increase in markers indicative of investment in short‐term survival. This is due to the high costs of male mating effort (Ellison, 2003) and the suggestion that survival may be prioritized over other processes (Bronson, 1991).

## 2 MATERIALS AND METHODS

Self‐reported heterosexual male athletes (total of 66) were recruited at the 2013 North Downs Way 100 (102.6 miles) race, held 10–11^th^ August (http://www.centurionrunning.com/north-downs-way-100-2015/). Athletes received an email explaining the study prior to race day, and were invited to participate. The study was approved by the University of Cambridge Human Biology Research Ethics Committee. The race is a relatively high profile event, with course records of 15:44:39 and 20:10:39 (hr:min:s) for men and women, respectively. Participants had a mean age of 29.4 years (range 20–37 years), and all were of European descent.

Samples and measurements were taken both before and after the race from athletes who completed the full race distance in order to determine the effects of the energetic deficit caused by race participation. Measurements included height, weight, and lean body mass (via bioelectrical impedance analysis). Five milliliters of saliva and 5 mL blood serum samples were collected for testosterone measurement and bacteria killing and hemolytic complement assays. While all of the prerace measurements were taken between 1800 and 2200 hours, the practicalities of varying finish times (ranging from 22:30 hours on the day of race start to 12:00 hours the following day) meant that postrace measurements were not standardized by time. All postrace measurements were collected within 15 minutes of completion of the race course following established methods (Berg et al., 2008; Davies & Thompson, 1986; Lucas et al., 2008; Malarkey et al., 1993; Stuempfle, Nindl, & Kamimori, 2010).

### 2.1 Reproductive effort

Testosterone was measured from saliva, rather than serum. Salivary analysis facilitates repeated sampling, enhances subject compliance, and provides reliable data (Dabbs, 1990; Shankar & Dandekar, 2012). Salivary measurements may underestimate testosterone levels; however, this is more problematic in females, and this study sought to track changes in testosterone levels within subjects rather than absolute differences between individuals (Shirtcliff, Granger, & Likos, 2002).

A 5 mL saliva sample was collected in multiple aliquots using the Salimetrics Saliva Collection Aid (#5016.02). Subjects refrained from eating, drinking, chewing gum, or brushing teeth in the 30 minutes preceding saliva collection. Cotton/polyester swabs were avoided and participants were screened for mouth injuries such as open sores to prevent blood contamination. Samples were immediately stored at −18°C, and were frozen at −80°C within 48 hours of collection. All samples were analyzed within two weeks of collection, using the DRG Salivary Testosterone ELISA kit (SLV‐3013). Prerace and postrace hormone levels were tested for normality, which was confirmed. Intra‐assay CV = 6.4%, inter‐assay CV = 10.26%. The high and low controls were within established values.

### 2.2 Lean body mass

Lean body mass was measured using bioelectrical impedance analysis (BIA) (BodyStat Quadscan4000). It is appreciated that *in vivo* measurements cannot measure body composition directly, but rather make predictions from other physiological metrics. BIA was chosen because of its speed, simplicity, high precision and suitability for assessing short‐term changes in individuals (Johnson, Bolonchuk, & Lykken, 1985; Roubenoff, 1996; Wells & Fewtrell, 2006). In order to avoid the inherent problems of predicting total body water (TBW), regression equations for converting between impedance and TBW were avoided. Instead, the simple index of 1/impedance, which reliably predicts lean mass index (lean mass/height^2^), was used (Wells et al., 2007).

### 2.3 Libido

For the purposes of this study, the terms libido and sexual desire are used to refer to desire to partake of sexual activity (Levin, 1994). This attitude is viewed as being ever‐present on a continuous scale, and is responsible for inducing a desire for sexual activity upon stimulation (Levin, 1994). A measurement of libido was taken both before and after the ultra‐marathon race, using near‐nude female images as stimuli. Visual stimuli have been shown to be powerfully sexually provocative for men (Graziottin, 2000; Hietanen & Nummenmaa, 2011; Quiney, Ketsetzis, Earls, & Karamanoukian, 1996). All participants self identified as being heterosexual.

Before the race, subjects practiced using the Self‐Assessment Manikin (SAM, these practice scores were not analyzed—Crabbe, Smith, & Dishman, 2007), a pictogram exhibiting levels of affect on a nine‐point scale (Hodes, Cooke, & Lang, 1985; Lang, 1980). The SAM has gained popularity in such work as it is a low‐cost and relatively easy method for quickly determining affective response in many contexts (Bradley & Lang, 1994). Subjects were randomly divided into two groups. The first group was shown a 45‐photo slide show (slide show A) before the race and a different 45‐photo slide show (slide show B) after the race, and this order was reversed for the second group. Each slide show comprised 15 provocative near‐nude female images, and 30 International Affective Picture System (IAPS) images (15 positive valence, 15 negative valence), and were viewed under standardized, private, and relaxed conditions. The order of images within each slide show was pseudo‐random such that two photos from the same category (provocative near‐nudes, positive and negative) could be viewed consecutively (Smith, 2012). Immediately after each picture was viewed, the participant used the SAM to rate their valence and arousal experienced whilst viewing the picture. Athletes were not asked to rate the same image more than once, to avoid possible development of habituated responses (Freund, Langevin, & Zajac, 1974; Heiman, 1977; Julien & Over, 1984; Rosen, 1973; Rubin & Henson, 1976). See Appendix for further details of this protocol used.

### 2.4 Innate immune function

*In vitro* bacteria killing assays were used with serum to measure innate immunity. After a test‐run to optimize dilutions, serum was diluted 1:12 in L‐glutamine supplemented CO_2_ Independent Media (Gibco #18045). A single lyophilized *E. coli* pellet (MicroBiologics Epower Microorganisms #0483E7) was reconstituted in sterile phosphate buffered saline and then diluted into a working solution, which produced approximately 200–300 colonies per 20 μL of aliquot. Aliquots of bacteria working solution were added to diluted serum in a microcentrifuge tube, vortexed, and incubated for 30 minutes. After incubation, the samples were spread on trypticase soy agar plates (BD BBL #211043) in triplicate and incubated overnight at 37°C. The number of colonies on each plate the next day were counted, and the percent bacteria killed for each sample relative to a positive control (media and bacteria only) was calculated.

Serum was also used to measure the classical pathway of complement protein activity via a hemolytic complement assay (Demas et al., 2011; Sinclair & Lochmiller, 2000). Following test runs to optimize dilutions, serum was diluted 1:90 and 1:180 in dextrose gelatin veronal buffer (Lonza BioWhittaker #10‐539) and pipetted in duplicate onto a round‐bottom 96‐well plate. Sheep red blood cells (MP Biomedicals #55876) were washed in sterile phosphate buffered saline, and further diluted to 0.6% in veronal buffer. Anti‐sheep red blood cell antibodies (Sigma #S1389‐1VL) were diluted to 1:40 in veronal buffer. Both the diluted antibodies and sheep red blood cells were then added to each sample well, and the plate was vortexed. Following incubation for 1.5 hours at 37°C, the plate was centrifuged, and the supernatant transferred to a new 96‐well round‐bottom plate. The absorbance of this supernatant was read at 405 nm. Results of this assay are expressed as CH50 units, or the inverse of the dilution predicted to cause 50% hemolysis (Mayer, 1948).

### 2.5 Statistics

Paired samples t‐tests were performed to compare prerace and postrace metrics. Associations between baseline (prerace) metrics were explored using Pearson correlation analysis. All analyses were performed using SPSS v21, and significance set at <0.05. *N* values vary due to some athletes not wishing to participate in certain aspects of the study (e.g., blood testing).

## 3 RESULTS

### 3.1 Height and weight

There was a significant decrease in both height (*n* = 66, prerace height *M* =176.9 cm, postrace *M* = 176.4 cm, mean change = −0.508 cm; 95% confidence interval −.846 to −.169) and weight (*n* = 66, prerace *M* = 76.9 kg, postrace *M* = 75.1 kg, mean change = −1.83 kg; 95% confidence interval −2.30 to −1.37) during the race.

### 3.2 Testosterone

There was a significant decrease in salivary testosterone levels from before (*n* = 52, *M* =109.59 pg/mL) to after the race (*n* = 52, *M* = 97.61 pg/mL, mean change = −12.0 pg/mL; 95% confidence interval −14.9 to −9.03).

### 3.3 Impedance

There was a significant decrease in bioelectrical impedance from before (*n* = 66, *M* =496.80) to after the race (*n* = 66, *M* = 454.90, mean change = −41.9; 95% confidence interval −53.3 to −30.5).

There was a significant increase in 1/impedance (1/*z*) from before (*n* = 66, *M* = 0.00204) to after the race (*n* = 66, *M* = 0.00223, mean change = 0.000185; 95% confidence interval 0.000134 to 0.000235).

### 3.4 Response to visual stimuli

While there was a significant decrease in arousal scores from before (*n* = 52, *M* = 5.29) to after the race (*n* = 52, *M* = 5.04, mean change = –.248; 95% confidence interval −.389 to −.108), there was no change in either valence (*n* = 52, before *M* = 5.37, after *M* = 5.29, SD = 0.69, mean change = –.0722; 95% confidence interval −.168 to −.0236) or dominance (*n* = 52, before M = 5.19, after 5.16, SD = 0.67, mean change = –.0325; 95% confidence interval −.121 to −.0556).

Further investigation considered how the three photo subtypes (provocative, positive valence, and negative valence) contributed to the overall decrease in arousal in response to the entire 45‐photo slide show. This revealed a significant decrease in arousal scores in response to provocative near‐nude images from before (*n* = 52, *M* = 5.40) to after the race (*n* = 52, *M* = 4.89, mean change = –.501; 95% confidence interval −.782 to −.220). In contrast, there was no significant difference in the arousal scores in response to positive valence images (*n* = 52, before *M* = 5.19, after *M* = 5.10, mean change = –.0833; 95% confidence interval − .230 to .0637) or in response to negative valence images (*n* = 52, before *M* = 5.27, after *M* = 5.11, mean change = –.160; 95% confidence interval −.365 to .0445). The difference in the total arousal score before and after the race can therefore be explained by a decrease in arousal in response to the near‐nude provocative images.

### 3.5 Bacteria killing assay

There was a significant increase in bacterial killing ability from before (*n* = 34, *M* = 30.0 percent killing) to after the race (*M* = 41.0 percent killing, mean change = 11.0; 95% confidence interval .847 – 21.2)

### 3.6 Hemolytic complement assay

There was a significant increase in hemolytic complement from before (*n* = 34, 124.60 CH50) to after the race (*n* = 34, 139.40 CH50, mean change = 14.9; 95% confidence interval 2.41 − 27.3)

**Figure 1:**
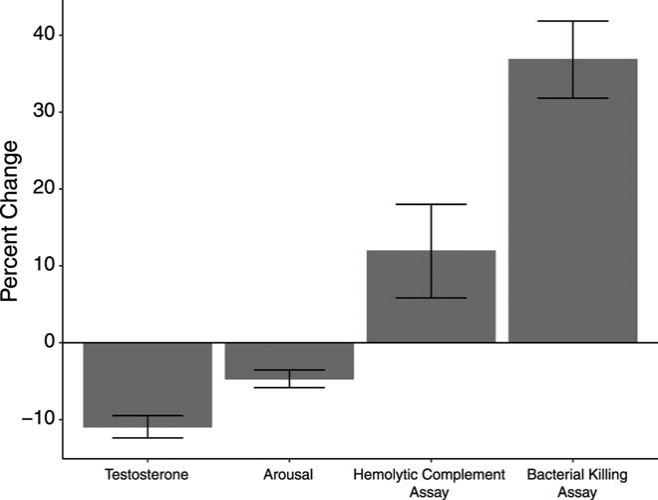
Bar chart showing percentage changes in metrics relating to reproduction (testosterone, arousal) and survival (haemolytic complement assay and bacteria killing assay)

These results are summarized in Table 1. Figure 1 shows percent change in measures of reproductive effort and immune function.

**Table 1:** Descriptive statistics for prerace and postrace metrics, with independent t‐test comparisons

	Prerace	Postrace	Comparison of prerace to postrace	
	Mean (SD)	Mean (SD)	*t*	*P*
Height (cm) (*n* = 66)	176.90 (6.69)	176.40 (6.53)	3.00	.004
Weight (kg) (*n* = 66)	76.90 (8.58)	75.10 (8.26)	7.91	< .001
Testosterone (pg/mL) (*n* = 52)	109.60 (17.60)	97.60 (17.50)	8.18	< .001
Impedance (*n* = 66)	496.80 (58.90)	454.90 (50.9)	7.32	< .001
Valence Total (*n* = 52)	5.36 (0.69)	5.29 (0.69)	1.51	.136
Arousal Total (*n* = 52)	5.29 (0.86)	5.04 (0.99)	3.55	.001
Dominance Total (*n* = 52)	5.19 (0.65)	5.16 (0.67)	0.74	.463
Haemolytic complement assay (CH50) (*n* = 34)	124.6 (30.20)	139.40 (33.30)	2.43	.021
Bacteria killing assay (% killing) (*n* = 34)	29.98 (36.70)	41.00 (39.60)	2.20	.035

### 3.7 Correlation analyses

Consideration of both baseline (prerace) and postrace metrics revealed a positive correlation between testosterone and total arousal (prerace *n* = 52, *r* = 0.33, *P* = .017; postrace *n* = 52, *r* = 0.35, *P* = .011). This result is unsurprising as testosterone has been implicated in sexual arousal.

The only other significant prerace correlations came between the subgroups of imagery contributing to the total arousal score, although there was a significant positive correlation between postrace impedance and the provocative imagery sub‐set of arousal scores (*n* = 52, *r* = 0.31, *P* = .025). We do not propose an explanation for this.

## 4 DISCUSSION

It was hypothesized that the energy deficit associated with ultramarathon participation would impose an energetic allocation trade‐off, causing an increased investment in short‐term survival (as measured by innate immune function) relative to reproductive effort. This hypothesis is supported by the observation of a significant increase in bacteria killing assay and hemolytic complement assay metrics, and a decrease in testosterone and arousal scores.

Evidence from sports medicine and related fields generally support the findings of increased immune function following intense exercise. These findings include temporary increases in the pro‐inflammatory cytokines interleukin‐6 (IL‐6) and tumor necrosis factor‐α (TNF‐α), granulocyte colony stimulating factor (GCSF), monocyte chemoattractant protein 1 (MCP‐1), and other immunological factors (Nieman, 2012). Natural killer (NK) cell function and numbers increase following bouts of exercise as well (McFarlin, Hutchison, & Kueht, 2008; Shephard & Shek, 1994; Woods, Davis, Smith, & Nieman, 1999), and lysozyme, an important anti‐bacterial component of innate mucosal immunity, shows a significant increase in male runners after two hours of running (Costa, Fortes, Richardson, Bilzon, & Walsh, 2012). In endurance athletes of both sexes, both leukocyte and lymphocyte counts are increased after an hour of intense exercise (McFarlin et al., 2008). While there are some reports of declining immune measures after exercise (e.g., sIgA), these resuts could be due to a number of different parameters, including sample collection, hydration, and even environmental factors (reviewed in Bishop & Gleeson, 2009).

On the whole, however, strenuous exercise is associated with changes in several inflammatory mediators (e.g., IL‐6) which are released from injured and/or contracting muscle cells during intense and prolonged exercise (Petersen & Pedersen, 2005). This leads to local neutrophil and monocyte invasion and phagocytosis of debris from damaged myocytes (Evans & Cannon, 1991; Northoff, Weinstock, & Berg, 1994; Weight, Alexander, & Jacobs, 1991). Changes in core body temperature have also been shown to affect leukocyte and neutrophil counts during bouts of exercise, although this effect is also mediated by environmental temperature (Mestre‐Alfaro et al., 2012; Sureda et al., 2015). Given the systemic effects of many immune molecules, it may be that localized increases induce or contribute to a general increase in immune function, as measured here. However, because these same molecules and cells may also be involved in minimizing or repairing the damage caused by intense physical exertion (Tidball, 2005), we maintain that this represents an additional investment in somatic maintenance, rather than a confounding factor. Furthermore, measuring multiple aspects of immune function (assays which do not measure inflammation, i.e., complement activity and bactericidal capabilities) may mitigate the effects of locally produced immune mediators on our results, as different immune responses are often driven by different mechanisms.

Although human males do not face the same energetic challenges as females (i.e., gametogenesis, gestation, and lactation), and energetic investment in spermatogenesis is minimal (Bagatell & Bremner, 1990; Elias, 1992), mating effort is still metabolically demanding. Energy costs include competition and mate attraction, as well as protection of and provisioning for mates and offspring (Muehlenbein & Bribiescas, 2005). While male mating effort may therefore be considered as largely behavioral, it does have important physiological correlates stemming from the need to signal underlying quality (Ellison, 2003; Zahavi, 1975).

The Immunocompetence Handicap Hypothesis (Folstad & Karter, 1992), which builds upon Zahavi's (1975) handicap hypothesis for the evolution of secondary sexual characteristics, suggests that testosterone mediates signals of reproductive status and quality in humans and other animals. In humans, muscle mass provides one such signal (Griggs et al., 1989; Kadi, 2008). Muscularity provides benefits in sexual selection from both inter‐ and intra‐sexual perspectives (Bribiescas, 2001; Dijkstra & Buunk, 2002; Frederick & Haselton, 2007; Gallup, White, & Gallup, 2007; Lavrakas, 1975). However, skeletal muscle mass accounts for approximately 20% of human male basal metabolic rate (Elias, 1992). While this figure is lower than the percentage of body mass accounted for by muscle (42% for a male, 36% for a female on average) (Marieb & Hoehn, 2010), suggesting that muscle may be a relatively cheap tissue at rest, it is the large size of some muscle groupings and high rates of energy consumption during activation that ensure muscles comprise a significant portion of the human energy budget (McArdle, Katch, & Katch, 2001). Skeletal muscle is therefore an expensive tissue to maintain and thereby constrains the amount of energy available for competing physiological functions such as immune responses (Muehlenbein & Bribiescas, 2005). Thus, a highly muscular phenotype, which is dependent on the anabolic effects of testosterone (Bhasin et al., 1996; Tsai & Sapolsky, 1996), presents an energetic handicap.

Additionally, high levels of testosterone have a number of other detrimental health effects, including increased incidence of prostate cancer, oxygen radical production, reduced tissue and organ maintenance, and injury associated with aggressive confrontational behavior (Lassek & Gaulin, 2009; Muehlenbein, 2006). As such, testosterone is an effective mediator of quality signalling due to the significant metabolic costs of a muscular physique (Andersson, 1994; Graffen, 1990; Zahavi, 1975), immunomudulation (Muehlenbein & Bribiescas, 2005; Muehlenbein & Watts, 2010; Muehlenbein, Cogswell, James, Koterski, & Ludwig, 2006), and the negative health effects of high levels of the hormone.

The high energetic demands associated with muscle mass mean that during periods of energetic deficits there may be a suppression of testosterone levels. This leads to reduced somatic reproductive effort through decreasing muscle mass (Bribiescas, 2001) and frequency of behaviors associated with mating. This study has demonstrated a decrease in electrical impedance. Although this could be in part due to reductions in hydration (Talluri, Lietdke, Evangelisti, Talluri, & Maggia, 1999), it is suggested that a breakdown of muscle tissue also contributed to these measurments (Friedl et al., 1994).

Our findings of both increased innate immunity and decreased testosterone levels in runners lends further support to the considerable literature on relationships between testerone and immune function (Bouman, 2005).

This study demonstrated an acute‐level trade‐off between reproduction and survivorship in ultramarathon runners. Because this is a highly trained and physically active group, these precise results may not be generalizable to the wider population. Such conditioning may buffer our participants from any negative health consequences of such strenuous exercise and nutritional imbalance. On the other hand, it could be argued that endurance running has cross‐cultural ecological relevance due to its importance in activities such as persistence hunting, as observed in the Kalarhari in Africa and the Tarahumara tribe of Northern Mexico (Liebenberg, 2006; Pennington, 1963). Indeed, endurance running may have played a role in our evolution (Carrier, 1984; Lieberman, Bramble, Raichlen, & Shea, 2009; Longman et al., 2015). We therefore maintain that results in different populations should be broadly comparable, given the universality of general life history theory predictions. Although we have used two immune measures, we have measured only one arm of the immune system, which is not indicative of total immunocompetence. Future avenues of research should include adaptive immune measures and use similar approaches to consider trade‐offs between other life history traits (i.e., growth). In addition to controlling for both core and environmental temperature, repeated postrace measures at different intervals could also help distinguish the precise contribution of muscle‐produced immunological mediators on increased immune function.

## CONFLICT OF INTEREST

The authors declare no conflict of interest.

## AUTHOR CONTRIBUTIONS

DL, JTS and JCKW designed the study as a whole, with further input from IDS, and DL collected the data. MPM contributed financially to laboratory analyses, and oversaw the laboratory analyses performed by ECS and SPP. DL performed statistical analyses, and all authors edited the manuscript.

## Appendix 1

Changes in general arousal that may be elicited by such prolonged exercise were controlled for, using the International Affective Picture System (IAPS) (Lang, Ohman, & Vaitl, 1988; Lang et al., 2005; Lang, Bradley, and Cuthbert, 2008). The IAPS is a large collection of color photographs designed for distribution and research as standardized affective materials. The system includes normative ratings of each photograph with respect to valence (pleasure), arousal, and dominance.

A pilot study of 30 subjects (age‐ and sex‐matched to race athletes) was conducted to collect SAM‐rated valence and arousal data on 30 provocative near‐nude female photographs. These photographs were obtained from a dating website, where visitors had given scores out of 10 to each image. The photographs chosen for the study had a mean score of 8.0 from a sample size of over 2000 website visitors. The pilot study enabled a determination of mean valence (6.45), arousal (5.11) and dominance (5.64). These thirty images were evenly split to make two groups, with equal mean valence, arousal, and dominance (see Table A1 below).

**Table A1:** Average valence, arousal and dominance scores of each image subset

	Prov A	Prov B	*t* (*P*)
Valence	6.49 (0.516)	6.41 (0.739)	0.315 (0.185)
Arousal	5.19 (0.712)	5.02 (0.641)	0.692 (0.420)
Dominance	5.47 (1.070)	5.81 (0.840)	−0.947 (0.192)

Thirty non‐sexual positive IAPS images were then selected to match the pilot‐derived arousal, valence, and dominance scores as closely as possible. These thirty images were split to make two groups of fifteen (Group A numbers: 1500, 1560, 2075, 2155, 2160, 8090, 5330, 5890, 8220, 7250, 7280, 7291, 7440, 7499, 8021; Group B numbers: 1540, 1590, 2152, 2158, 2224, 8208, 5628, 5990, 8467, 7279, 7289, 7402, 7450, 7660, 8060).

A further thirty non‐sexual negative IAPS images were selected with matched arousal and dominance, but with a negative valence (3.39). Again, these thirty images were split to make two groups of fifteen (Group A numbers: 1220, 1274, 2055.1, 2120, 2683, 2692, 2710, 3181, 3212, 3300, 6021, 6200, 6561, 6836, 9101; Group B numbers: 1111, 1275, 2039, 2457, 2691, 2694, 2717, 3160, 3213, 3301, 6022, 6250.1, 6570, 6834, 9102). This design allowed the possibility of detecting any effects the exercise may have on the arousal valuations of both positive and negative pictures. Table A2 shows the average valence, arousal and dominance scores of each image subset.

**Table A2:** Mean (standard deviation) valence, arousal and dominance for each image subset, independent *t*‐test (*P*‐value) reported no significant difference between the image subsets

	Prov A	Prov B	*t* (*P*)	IAPS+ A	IAPS+ B	*t* (*P*)	IAPS‐ A	IAPS‐ B	*t* (*P*)
Valence	6.49 (0.516)	6.41 (0.739)	0.315 (0.185)	6.54 (0.223)	6.56 (0.238)	−0.332 (0.742)	3.45 (0.431)	3.33 (0.607)	0.590 (0.560)
Arousal	5.19 (0.712)	5.02 (0.641)	0.692 (0.420)	5.15 (0.502)	5.07 (0.415)	0.503 (0.619)	5.10 (0.600)	5.20 (0.956)	−0.352 (0.727)
Dominance	5.47 (1.07)	5.81 (0.840)	−0.947 (.192)	5.65 (0.927)	6.01 (0.481)	−1.298 (0.205)	4.85 (0.552)	5.01 (0.753)	−0.675 (0.506)

The valence, arousal and dominance scores for the 2 subsets of provocative images (abbreviated below) were obtained through a pilot study.

Independent samples t‐tests have confirmed that there were no significant differences between slide show A and slide show B in any of the three metrics (valence, arousal, or dominance). Furthermore, Levene's Test confirmed there were no significant differences in the variances of any variable.
